# Diagnostic Value of Endotracheal Aspirates Sonication on Ventilator-Associated Pneumonia Microbiologic Diagnosis

**DOI:** 10.3390/microorganisms5030062

**Published:** 2017-09-20

**Authors:** Laia Fernández-Barat, Ana Motos, Otavio T. Ranzani, Gianluigi Li Bassi, Elisabet Aguilera Xiol, Tarek Senussi, Chiara Travierso, Chiara Chiurazzi, Francesco Idone, Laura Muñoz, Jordi Vila, Miquel Ferrer, Paolo Pelosi, Francesco Blasi, Massimo Antonelli, Antoni Torres

**Affiliations:** 1 Centro de Investigación Biomedica En Red-Enfermedades Respiratorias (CibeRes, CB06/06/0028), Institut d’Investigacions Biomèdiques Agust Pi i Sunyer (IDIBAPS), 08036 Barcelona, Spain; amotos@clinic.cat (A.M.); otavioranzani@yahoo.com.br (O.T.R.); glibassi@clinic.cat (G.L.B); eli_agui@hotmail.com (E.A.X.); miferrer@clinic.cat (M.F.); 2 Department of Medicine, School of Medicine, University of Barcelona, 08036 Barcelona, Spain; 3 Pulmonary and Critical Care Unit, Respiratory Institute, Hospital Clinic, 08036 Barcelona, Spain; 4 Dipartimento Scienze Chirurgiche e Diagnostiche Integrate (DISC), Università degli Studi di Genova, 16132 Genova, Italy; t.senussi@hotmail.it (T.S.); ppelosi@hotmail.com (P.P.); 5 Respiratory Unit, Ospedale Garbagnate Salvini, Azienda Socio Sanitaria Territoriale Rhodense, 20024 Garbagnate Milanese, Italy; chiara_travierso@yahoo.it; 6 Department of Anesthesiology and Intensive Care Medicine, Humanitas Clinical and Research Center, Via Manzoni 56, 20089 Rozzano, Milano, Italy; chiara.chiurazzi@gmail.com; 7 Department of Anesthesiology and Intensive Care Medicine, Università Cattolica del Sacro Cuore, Fondazione Policlinico Universitario Agostino Gemelli, 00135 Rome, Italy; axl38@hotmail.com (F.I.); Massimo.Antonelli@Unicatt.it (M.A.); 8 Barcelona Institute for Global Health (ISGlobal) Hospital Clínic, 08036 Barcelona, Spain; laura.munoz@isglobal.org (L.M.); jvila@clinic.cat (J.V.); 9 Department of Basic Clinical Practice, School of Medicine, University of Barcelona, 08036 Barcelona, Spain; 10 Department of Pathophysiology and Transplantation, Università degli Studi di Milano, 20122 Milan, Italy; francesco.blasi@unimi.it; 11 Department of Internal Medicine, Respiratory Unit and Cystic Fibrosis Adult Center, Fondazione IRCCS Cá Granda, Ospedale Maggiore Policlinico, 20122 Milan, Italy

**Keywords:** biofilm, endotracheal aspirate, sonication, ventilator associated pneumonia, animal model, *Pseudomonas aeruginosa*, *Staphylococcus aureus*

## Abstract

Microorganisms are able to form biofilms within respiratory secretions. Methods to disaggregate such biofilms before utilizing standard, rapid, or high throughput diagnostic technologies may aid in pathogen detection during ventilator associated pneumonia (VAP) diagnosis. Our aim was to determine if sonication of endotracheal aspirates (ETA) would increase the sensitivity of qualitative, semi-quantitative, and quantitative bacterial cultures in an animal model of pneumonia caused by *Pseudomonas aeruginosa* or by methicillin resistant *Staphylococcus aureus* (MRSA). Material and methods: *P. aeruginosa* or MRSA was instilled into the lungs or the oropharynx of pigs in order to induce severe VAP. Time point assessments for qualitative and quantitative bacterial cultures of ETA and bronchoalveolar lavage (BAL) samples were performed at 24, 48, and 72 h after bacterial instillation. In addition, at 72 h (autopsy), lung tissue was harvested to perform quantitative bacterial cultures. Each ETA sample was microbiologically processed with and without applying sonication for 5 min at 40 KHz before bacterial cultures. Sensitivity and specificity were determined using BAL as a gold-standard. Correlation with BAL and lung bacterial burden was also determined before and after sonication. Assessment of biofilm clusters and planktonic bacteria was performed through both optical microscopy utilizing Gram staining and Confocal Laser Scanning Microscopy utilizing the LIVE/DEAD^®^*Bac*Light kit. Results: 33 pigs were included, 27 and 6 from *P. aeruginosa* and MRSA pneumonia models, respectively. Overall, we obtained 85 ETA, 69 (81.2%) from *P. aeruginosa* and 16 (18.8%) from MRSA challenged pigs. Qualitative cultures did not significantly change after sonication, whereas quantitative ETA cultures did significantly increase bacterial counting. Indeed, sonication consistently increased bacterial burden in ETAs at 24, 48, and 72 h after bacterial challenge. Sonication also improved sensitivity of ETA quantitative cultures and maintained specificity at levels previously reported and accepted for VAP diagnosis. Conclusion: The use of sonication in ETA respiratory samples needs to be clinically validated since sonication could potentially improve pathogen detection before standard, rapid, or high throughput diagnostic methods used in routine microbial diagnostics.

## 1. Introduction

Ventilator associated pneumonia (VAP) may occur after 48 h of oro-tracheal intubation with a pooled mean incidence of 10 episodes for 1000 ventilator-days, becoming one of the principal Intensive Care Unit (ICU)-acquired infections worldwide [1,2,3]. Accurate and rapid diagnostic methods are key to initiate appropriate antimicrobial treatment and to reduce VAP relapse, healthcare costs, mortality, and an indirect effect on the emergence of bacterial resistance [4,5,6]. A wide new panel of rapid diagnostic technologies offers promising possibilities for the optimization of antibiotics usage [7]. However, adequate implementation of these novel technologies is an important consideration as they also present limitations. A notable limitation of these technologies is that they provide an important amount of raw data that requires qualified interpretation before clinical decision making. Furthermore, new unexpected limitations may emerge as these new technologies become routinely implemented.

Current strategies for microbiological diagnosis of VAP include the microbiological culture of one of the following respiratory samples: endotracheal aspirate (ETA), bronchoalveolar aspirate (BAS), bronchoalveolar lavage (BAL), and protected specimen brush (PSB). The cut-off for bacterial growth to differentiate between colonization and infection are 5 or 6 log CFU/mL for ETA and BAS, 4 log CFU/mL for BAL, and 3 log CFU/mL for PSB [7,8,9]. BAL is probably the most representative respiratory sample for diagnosing VAP. The reason for this is not only the extensive area of alveoli explored, but also the quality of the samples obtained. This allows the detection of intracellular organisms, rapid molecular techniques, rapid stains, and qualitative or quantitative cultures [10]. The use of ETA instead of BAL has been associated with VAP over-diagnosis, without any impact on VAP clinical outcomes [9,11,12,13]. ETA and BAL cultures offer good positive and negative predictive values for VAP diagnosis when utilized before antibiotics administration or change of treatment. Nevertheless, differentiation between colonization and infection remains a limitation of both semi-quantitative cultures and rapid diagnostic tools. An integrated approach that balances clinical judgment and microbiological results is likely the best approach for VAP diagnosis and treatment [14].

There is consistent evidence that microorganisms can grow in biofilms within respiratory secretions, because mucus stimulates biofilm production [15,16,17]. Biofilm associated infections (BAI) are of great clinical concern because the biofilm’s mode of growth is responsible for culture negative results, recalcitrance to antimicrobial treatment, and emergence of antimicrobial resistance [18,19]. Therefore, it is challenging to implement diagnostic tools without misleading diagnostics of BAI. In a landmark study, Trampuz et al. (2007) demonstrated that sonication of the sample significantly increased the sensitivity and specificity of bacterial cultures for diagnosis of biofilm-associated prosthetic joint infections [20]. Sonication of fluids containing the sample was recommended in the latest clinical guidelines for the treatment and management of BAI, especially for catheter and prosthetic related infections [21]. However, studies are lacking on the utility of sonication of respiratory samples to improve VAP diagnosis.

Recent investigations have demonstrated the presence of biofilm aggregates in BAL samples from children with non-cystic fibrosis bronchiectasis by confocal laser scanning microscopy (CLSM) [22]. We previously demonstrated the presence of biofilm aggregates growing directly attached to the internal surface of endotracheal tubes (ETT) and/or associated within host respiratory secretions in an animal model of either methicillin resistant *Staphylococcus aureus* (MRSA) or *Pseudomonas aeruginosa* [23,24].

We hypothesized that sonication of the ETA would increase bacterial release from biofilm aggregates, thus improving the sensitivity and specificity of VAP microbiological diagnosis. In this study, we compared bacterial qualitative, semi-quantitative, and quantitative cultures before and after ETA sonication in an animal model of pneumonia, to determine sonication’s effect on the sensitivity and specificity of bacterial cultures and any improvement between ETA and lung bacterial growth correlation after ETA sonication.

## 2. Material and Methods

### 2.1. Population

All endotracheal aspirates from several ongoing porcine studies of VAP and severe pneumonia caused by either *Pseudomonas aeruginosa* or MRSA were included.

*P. aeruginosa* or MRSA cultures were instilled into the lungs or the oropharynx to induce severe or ventilator-associated pneumonia in anesthetized, orotracheally intubated, and mechanically ventilated pigs, respectively [25,26]. Both models have been previously validated by our group and the primary difference is that the bronchial challenge rapidly develops severe pneumonia whilst the oropharynx instillation closely mimics the pathophysiology of VAP [25,26,27]. Briefly, in the first model, 75 mL of 7 log CFU/mL of a log-phase culture (Luria Broth (LB), OD_600nm_ = 0.1–0.2) was instilled into the lungs, whereas in the second model, 5 mL of 7 log CFU/mL of a log-phase culture was instilled twice into the oropharynx. Animals were kept mechanically ventilated and were euthanized seventy-two hours after bacterial challenge. The institutional review board and animal ethics committee approved all included studies. The project license number that covered the animal experiments was the following: 06/17. 9322 (start date 14/12/16 and the expiration date 28/03/19). Animals were managed according to the National Institutes of Health guidelines for the Use and Care of Animals. Additional details on animal handling and methods are reported in previous publications [26].

### 2.2. Collection of Samples

Paired ETA and BAL were obtained sequentially at 24, 48, and 72 h after bacterial instillation to perform qualitative, semi-quantitative, and quantitative bacterial cultures in each animal. Tracheal suctioning was performed using a 12-Fr standard CSS (KIMVENT* Closed Suction Systems, Kimberly Clark, Irving, TX, USA), as clinically recommended [28]. BAL was performed using a bronchoscope (Pentax SAFE-3000; Ricoh Imaging Deutschland GmbH, Hamburg, Germany) in the right medium lobe with two 10-mL aliquots of sterile saline solution. The first aliquot was discarded, while the second one was used for quantitative microbiology studies. Pigs were euthanized after 72 h of bacterial challenge and lung tissue samples were harvested to assess quantitative cultures.

### 2.3. Microbiological Analysis

ETA and BAL were liquified in sterile 0.9% saline solution (NaCl), homogenized with a vortex mixer and then serially diluted by aseptic transfer of 0.1 mL samples into 0.9 mL of sterile 0.9% NaCl solution to yield dilutions of 10^−1^ to 10^−3^-fold on blood and MacConkey agar and incubated at 37 °C over-night.

Two-hundred µL of each ETA sample were needed to perform standard cultures, whilst the remaining volume of sample (minimum 200 µL) was sonicated in ultrasonic cleaning equipment (Branson 3510 E-MT; Bransonic, Danbury, CT, USA) for 5 min at 40 KHz to disaggregate biofilms, before undergoing serial dilutions and plating as aforementioned. Bacterial growth was quantified and reported as log_10_ colony-forming units per milliliter (log CFU/mL). Microorganisms were identified by mass spectrometry through a Microflex LT (Bruker Daltonik GmbH, Bremen, Germany) benchtop instrument controlled by the FLEXCONTROL software (version 3.0; Bruker Daltonics). Spectra were analysed with the MALDI BioTyper software (version 3.1; Bruker Daltonics) using the pre-processing and Bio- Typer main spectrum (MSP) identification standard methods (mass range 2000–20,000 *m*/*z*) against the default Bruker database. Accuracy of the identification was determined by a loga-rithmic score value resulting from the alignment of peaks to the best matching reference spectrum. All bacterial quantitative cultures were performed in duplicates.

Five tissue samples (80–120 mg each) from the five lobes of the lungs were excised and placed on sterile vials. Lung biopsies were aseptically homogenized by using a glass tissue mortar, in a volume of 0.9% NaCl solution to yield a 1:5 (*w*/*v*) suspension of ground tissue.

### 2.4. Microscopy Images Acquisition

To qualitatively assess biofilm clusters and planktonic bacteria, standard Gram staining and confocal laser scanning microscopy (CLSM) were performed for both non-sonicated and sonicated ETA samples. For Gram staining, the conventional timing and dies were used. Gram-stained slides were inspected using a 100× oil objective on a DMRB microscope with equipped with a color camera.

ETA samples were immediately stained after extraction with the LIVE/DEAD^®^*Bac*Light kit™ (*Bac*Light kit™, Invitrogen, Barcelona, Spain), adding 1.5 µL SYTO9 and 1.5 µL popidium iodide (PI) dyes in 1 mL PBS 1× during 15 min in the dark. Then, a very thin layer was smeared onto the slide. Images were obtained with a Leica TCS SP5 laser scanning confocal system (Leica Microsystems Heidelberg GmbH, Manheim, Germany) equipped with a DMI6000 inverted microscope, and a 63× PL APO numerical aperture 0.7 oil immersion objective was used. SYTO^®^ 9 (Invitrogen, Barcelona, Spain) and PI images were acquired sequentially using 488, 561 nm laser lines, AOBS as beam splitter, and emission detection ranges 500–550, 570–620 nm, respectively. The confocal pinhole was set at 1 Airy unit and pixel size was 160 nm.

### 2.5. Statistical Analysis

Categorical variables are reported in percentage (%) and continuous variables as mean ± SD if normally distributed. Paired samples were compared using the paired *t*-test. Spearman correlation coefficient analyses were performed to determine associations between quantitative variables. For the correlation between ETA and lung tissue burden, we selected samples from animals that did not receive nebulized antibiotics, because of the extreme high antimicrobial activity in the ETA samples. The sensitivity, specificity, and positive and negative predictive value of microbiologic cut-offs were calculated with two-by-two contingency tables using standard formulae, when paired ETA and BAL samples were available. All statistical analyses were performed using IBM SPSS Statistics version 22.0 (Armonk, NY, USA). Two-tailed testing was used and *p* < 0.05 was considered statistically significant.

## 3. Results

### 3.1. Population

We analyzed thirty-three pigs, 27 and 6 belonged to *P. aeruginosa* and MRSA pneumonia models, respectively. Twenty-four pigs were intrabronchially challenged with either *P. aeruginosa* or MRSA and included in studies to test pneumonia treatments. In contrast, nine pigs were challenged with the bacterial suspension instilled into the oropharynx for VAP preventive studies (Table 1).

### 3.2. Samples Collected

Overall we obtained 55 BAL and 85 ETA, 69 (81%) from *P. aeruginosa* versus 16 (19%) from MRSA challenged pigs, 61 (72%) belonged to pigs intrabronchially challenged versus 24 (28%) obtained from the oropharynx instilled model. Among the 85 ETA samples: 33, 25, and 27 were obtained at 24, 48, or 72 h (autopsy) after bacterial challenge, respectively. A total of 36 versus 49 ETA samples were obtained from untreated versus treated pigs, respectively (Table 1).

### 3.3. Standard Versus Sonicated Cultures

**Figure 1:**
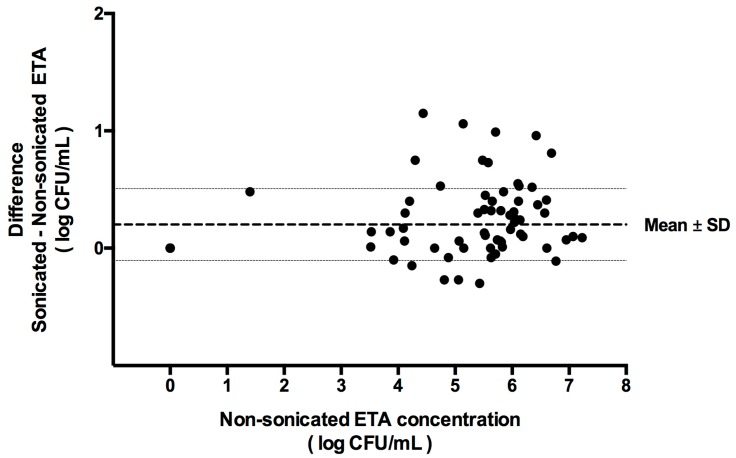
Bland–Altman plot showing the differences between sonicated and non-sonicated endotracheal aspirate (ETA) paired samples (Mean ± SD). Eighty-one quantitative ETA cultures were performed twice: before and after sonication. Each single dot represents the difference between bacterial burden of sonicated minus non-sonicated ETA, using non-sonicated values in the *X* axis. Of notice, 52 out of 81 (64%) dots were allocated above zero, 21 out of 81 (26%) at zero, and 8 out of 81 (10%) below zero. Mean difference ± SD was 0.2 ± 0.3 log CFU/mL (*p* < 0.001).

No differences were found on the qualitative or semi-quantitative VAP microbial diagnosis when comparing sonicated versus non-sonicated ETA samples, independent of whether the cut-off used was 5 or 6 log CFU/mL. In contrast, in 81 ETA quantitative bacterial cultures (4 were uncountable) we found that bacterial burden (log CFU/mL) was significantly higher in sonicated versus non-sonicated ETA cultures (4.42 ± 2.58 vs. 4.18 ± 2.5 respectively; *p* < 0.001) (Figure 1).

In particular, we analyzed 69 vs. 16 ETA samples obtained from *P. aeruginosa* vs. MRSA models, respectively. Sonicated ETA samples presented higher *P. aeruginosa* burden versus non-sonicated ones. However, the increase in MRSA burden after sonication did not reach statistical significance (Table 2).

Along time assessments, sonicated vs. non-sonicated ETA consistently presented higher bacterial burden at 24, 48, and at 72 h after bacterial challenge (Table 2).

Sonication also increased ETA bacterial burden when the bacterial challenge was intrabronchial. In contrast, no difference in bacterial burden was found between sonicated and non-sonicated quantitative cultures for the 9 pigs that received bacterial instillation of the oropharynx (Table 2).

### 3.4. Sensitivity and Specificity

The sensitivity (Se) and the specificity (Sp) of sonicated and non-sonicated quantitative cultures of ETAs used for the diagnosis of pneumonia were determined using BAL ≥ 4 log CFU/mL as a gold standard. The Se/Sp analysis were performed for both ETA cut-off points: ≥5 log CFU/mL and ≥6log CFU/mL. No improvement in Se/Sp was detected after ETA sonication when the ETA cut-off was ≥5 log CFU/mL. In contrast, Se improved from 75%, in non-sonicated samples, to 87.5% after sonication. Additionally, Sp decreased from 83 to 70.2% when the ETA cut-off was ≥6 log CFU/mL (Table 3).

**Figure 2:**
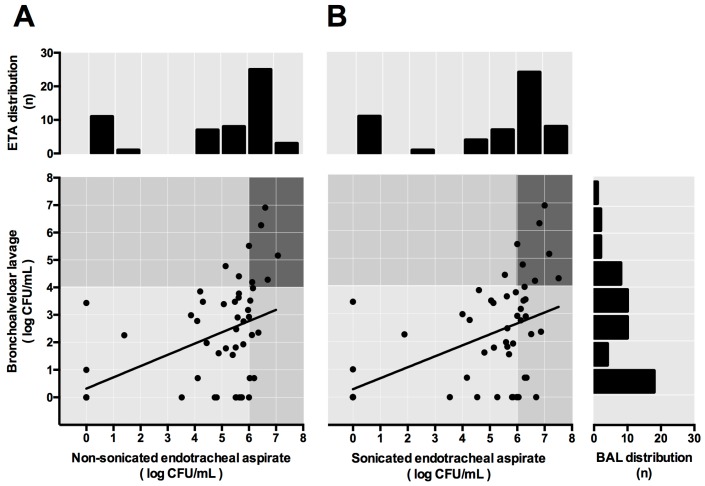
Correlation between quantitative cultures of ETA and BAL for non-sonicated (**A**) and sonicated samples (**B**). The histograms represent the number of ETA (on the top) or BAL (right) on each bacterial concentration. Moderate correlation was found between non-sonicated and sonicated ETA and BAL(*r* = 0.51 and *r* = 0.56, respectively; *p* = 0.75). ETA and BAL cut-off was 6 log CFU/mL and 4 log CFU/mL, respectively. After sonication the number of true positives samples increased from 6 (11%) to 7 (13%) out of 55 samples.

**Figure 3:**
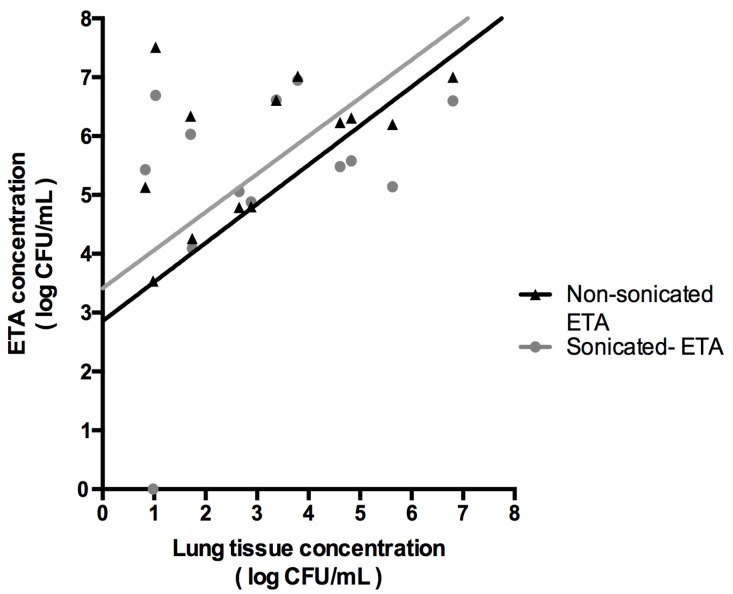
Correlation between ETA and lung tissue bacterial burden. Of note, in 15 cases a moderate correlation was found between non-sonicated or sonicated ETA and lung tissue bacterial burden (*r* = 0.52 and *r* = 0.57, respectively; *p* = 0.86).

In 55 cases, a moderate correlation was found to exist between non-sonicated and sonicated ETA and BAL bacterial loads. However, the non-sonicated vs. sonicated ETA-BAL correlation did not significantly improve after sonication (*r* = 0.51 and *r* = 0.56, respectively; *p* = 0.75) (Figure 2). The correlation between non-sonicated vs. sonicated ETA and lung tissue bacterial load did not significantly improve after sonication either (*r* = 0.52 and *r* = 0.57, respectively; *p* = 0.86), although this last analysis included only 15 cases (Figure 3).

### 3.5. Imaging Biofilms

**Figure 4:**
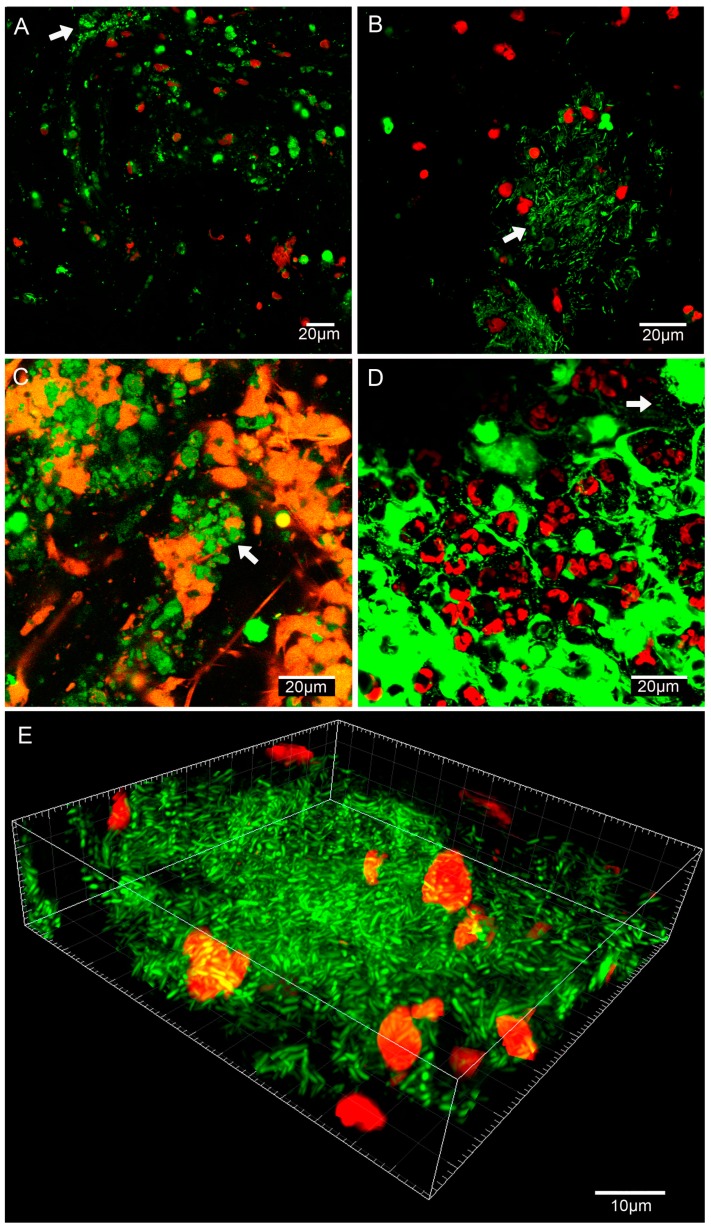
Confocal Laser Scanning Microscopy (CLSM) image of ETA samples before and after sonication in *P. aeruginosa* and MRSA infection models. Of note, viable bacteria (in green) stained with the SYTO 9, a green Fluorescent Nucleic Acid Stain, were visible. Dead bacteria (in red) stained with the propidium iodide (PI) were barely detected. The nucleus and cytoplasm of eukaryotic cells from the pig were also stained nonspecifically with the PI and SYTO 9 (large red and green blotches). (**A**) vs. (**B**) correspond to non-sonicated vs. sonicated ETA (5.14 vs. 6.20 log CFU/mL) after 72 h of *P. aeruginosa* instillation with biofilm clusters visible (white arrow). Similarly, (**C**) vs. (**D**) correspond to non-sonicated vs. sonicated ETA (1.38 vs. 1.88 log CFU/mL) after 72 h of MRSA instillation with biofilm clusters and free-floating cocci (white arrows), respectively. (**A**,**B**) images belong to a pig treated with IV cephalosporin and (**C**,**D**) to a pig treated with IV lipoglycopeptide. (**E**) 3D reconstruction of a Gram-negative bacilli biofilm cluster (Imaris, Bitplane, Oxford instruments Company, Abingdon, UK).

**Figure 5:**
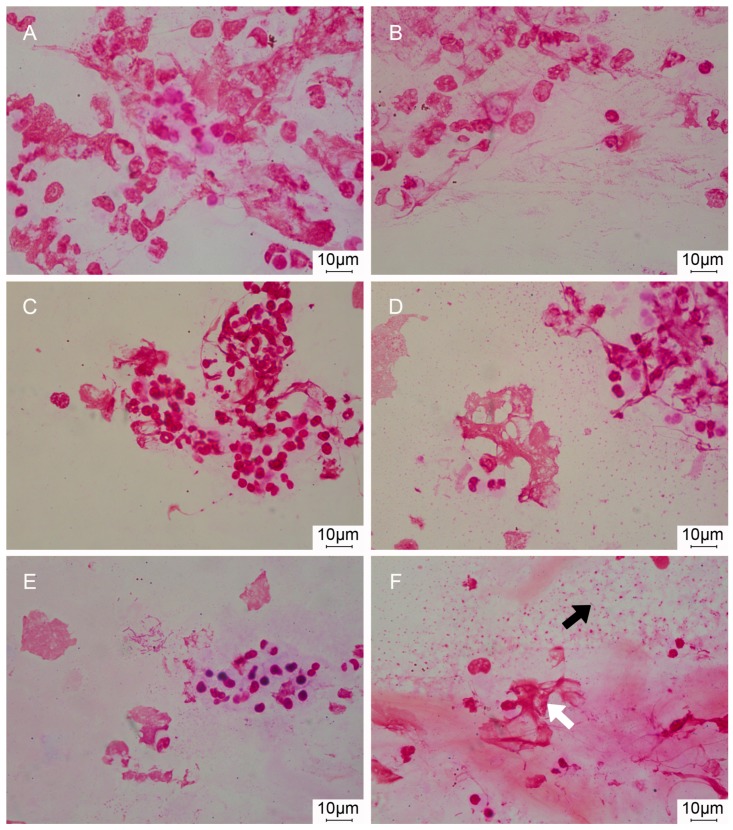
Optical microscopy images of Gram stained ETA before and after sonication. Images (**A**) vs. (**B**), (**C**) vs. (**D**) and (**E**) vs. (**F**) correspond to non-sonicated vs. sonicated ETA at 24, 48, and 72 h, respectively. Biofilm aggregates (white arrow) were observed before and after sonication. In contrast, free-floating bacteria were mainly detected after sonication (black arrow).

*P. aeruginosa* biofilm aggregates were identified by optical microscopy and CLSM before and after sonication (Figure 4). Increased amounts of free-floating bacteria were identified, particularly on Gram staining, after ETA sonication at 24, 48, and 72 h after bacterial challenge (Figure 5).

## 4. Discussion

Optimizing VAP diagnosis has become a top priority because of the increasing prevalence of multidrug resistant pathogens in ICUs and the scarce availability of new therapy options. In this context, it is crucial to have a rapid and accurate microbiological diagnosis in order to improve treatment guidance. Our study deals with the ability of microorganisms to form biofilms within respiratory secretions and highlights sonication as a method to disaggregate them before applying either standard, rapid, or high throughput diagnostic technologies in order to improve pathogen identification. Smooth sonication is the gold standard for disaggregating biofilms in sputum of cystic fibrosis patients [29,30], prosthetic joint devices [20], catheters [31,32], or endotracheal tubes [24,33]. In line with previous findings, we demonstrated that bacterial counts were higher in sonicated versus non-sonicated ETA samples, and this finding was consistent at different time points.

A BAL threshold ≥4 log CFU/mL has been used instead of histology as a gold standard [34] for the sensitivity and specificity analyses in previous studies [9]. We found 75% Se and 83% Sp of non-sonicated ETA, using the cut-off ≥6 log CFU/mL. Our Se/Sp values are higher than 54%/75% reported by Valencia et al. [8], but in line with those reported by Morris et al., or by Jourdain et al. using BAL samples [35]. Differences between studies could be influenced by the gold standard used or by sample size, which was superior in the Valencia et al study [8]. Interestingly, in our study, sensitivity was notably increased after sonication (87.5%) and specificity (70%) was similar [8] or even superior to other previous studies [9]. This increase in sensitivity was only apparent when the cut-off of ETA burden was ≥6 log CFU/mL. This stirs the debate about the most appropriate ETA cut-off. Those defending a lower cut-off point opt for greater sensitivity at the expense of losing specificity, and vice versa. Our results suggest that with a cut-off ≥6 log CFU/mL, ETA sonication grants higher sensitivity whilst maintaining high specificity. Subsequently, further investigations should prospectively investigate the utility of sonication in routine clinical diagnostics.

Further study is required to determine whether sonication is an effective adjunct for both *P. aeruginosa* and MRSA ETA cultures. Our previous results indicated that both *P. aeruginosa* and MRSA formed biofilms in ETTs and also in respiratory secretions accumulated within the ETT [23,24]. Interestingly, Hola V. and coauthors investigated the microbial composition in urinary catheters by applying sonication and found that it improved diagnosis of urinary tract infections, especially when strong biofilm producers were present [31]. We found that ETA sonication increased bacterial counting of *P. aeruginosa* but did not reach a statistically significant increase for MRSA growth. The reason for this could be either attributed to a better biofilm formation capability of *P. aeruginosa*, to a lower number of animals challenged with MRSA, or to a combination of both.

The latest clinical guidelines for the diagnosis and treatment of BAI strongly recommend the use of imaging techniques [21]. Recent investigations have evidenced the presence of biofilms by CLSM in BAL samples of children with non-cystic fibrosis bronchiectasis [22]. In the present study we confirmed that *P. aeruginosa* biofilms are also found in ETA samples from a pig model of pneumonia. Importantly, biofilms were identified by two independent microscopy techniques (Optical microscopy-Gram stain or CLSM-Live/Dead Kit) even after sonication. Our findings are relevant as they imply that we may be sub-optimally utilizing existing diagnostic technology if we do not disaggregate bacteria from their biofilms before ETA culture. Considering the latest advancements in point-of-care diagnosis [36] and that biofilm forming species of the respiratory microbiome can trigger pathogen growth [22,37,38], this seems the appropriate time to reconsider the diagnosis of biofilms in respiratory samples from intubated ICU patients with suspicion of VAP.

Several limitations should be addressed within our study. First, interestingly, the study comprised different pneumonia models that differed by the microorganism, the type of bacterial challenge, and the treatment administered. This was especially a limitation for sub-analyses as it decreased the sample size in specific cases. Second, BAL missing samples or the exclusion of lung tissue samples from pigs treated with nebulized antibiotics limited the Se/Sp and the power of the correlation analyses. Ultimately, the beneficial effects of sonication was only detectable in quantitative cultures, which are not the routine technique used for microbial diagnosis [39]. Nevertheless, our results provide enough evidence that *P. aeruginosa* biofilms are present in ETA samples, even after sonication, and this can influence the sensitivity of not only conventional, but also high throughput and rapid diagnostics.

## 5. Conclusions

Sonication of ETA improves the detection of *P. aeruginosa* within biofilms, improving sensitivity and maintaining specificity for VAP microbiological diagnosis in a pig model of pneumonia. Its utility in routine clinical laboratory or before rapid or high throughput technologies needs to be prospectively investigated.

## Figures and Tables

**Table 1:** Number of animals, microorganism, instillation model, and treatment of the different studies.

Study	Pigs (*n*)	Inoculated Microorganism	Bacterial Instillation	ETA (*n*)	BAL (*n*)	Treatment
Study 1	15	*P. aeruginosa*	Lungs	39	23	Untreated controls vs. Nebulized aminoglycoside vs. IV carbapenem
Study 2	4	MRSA	Oropharynx	10	0	Untreated controls vs. Monoclonal antibody
Study 3	7	*P. aeruginosa*	Lungs	16	14	Untreated controls vs. IV cephalosporin
Study 4	5	*P. aeruginosa*	Oropharynx	14	12	Untreated controls vs. Monoclonal antibody
Study 5	2	MRSA	Lungs	6	6	Untreated controls vs. IV lipoglycopeptide

IV: Intravenous; Dosages: aminoglycoside (300 mg/kg every 6 h), carbapenem (25 mg/kg every 8 h), cephalosporin (50 mg/kg every 8 h), Monoclonal antibody against *P. aeruginosa* (15 mg/Kg single dose), lipoglycopeptide (25 mg/kg every 24 h). Study 2 only comprised untreated pigs.

**Table 2:** Comparison of non-sonicated versus sonicated ETA bacterial load by microorganism, instillation model and time of assessment.

		Non-Sonicated ETA (log CFU/mL)	Sonicated ETA (log CFU/mL)	*p*-Value
**Inoculated microorganism**	*P. aeruginosa* (*n* = 69)	4.03 ± 2.58	4.25 ± 2.79	*p* < 0.001
	MRSA (*n =* 16)	4.82 ± 2.04	5.17 ± 1.52	0.159
**Bacterial instillation**	Lungs (*n* = 61)	4.10 ± 2.50	4.35 ± 2.65	*p* < 0.001
	Oropharynx (*n* = 24)	4.37 ± 2.53	4.61 ± 2.42	0.156
**Time of assessment** **(hours after bacterial challenge)**	24 h (*n* = 33)	5.24 ± 1.65	5.45 ± 1.71	*p* < 0.001
	48 h (*n =* 25)	3.67 ± 2.69	3.79 ± 2.82	0.019
	72 h (*n* = 27)	3.30 ± 2.84	3.62 ± 2.89	0.052

**Table 3:** Effect of ETA sonication on the diagnosis of pneumonia using BAL ≥ 4 log CFU/mL as gold standard.

	ETA cut-off	Se	Sp	PPV	NPV	PLR	NLR
		(95%CI)	(95%CI)	(95%CI)	(95%CI)	(95%CI)	(95%CI)
**Non-sonicated ETA**	≥5 log CFU/mL	100	46.8	24.2	100	1.88	Undefined
		(100–100)	(32.5–61.1)	(9.6–38.9)	(100–100)	(1.44–2.46)	
	≥6 log CFU/mL	75.0	83.0	42.9	95.1	4.41	0.30
		(65.6–100)	(72.2–93.7)	(16.9–68.8)	(88.5–100)	(2.09–9.3)	(0.09–1.01)
**Sonicated** **ETA**	≥5 log CFU/mL	100	40.4	22.2	100	1.68	Undefined
		(100–100)	(26.4–54.5)	(8.6–35.8)	(100–100)	(1.33–2.12)	
	≥6 log CFU/mL	87.5	70.2	33.3	97.1	2.94	0.18
		(64.6–100)	(57.1–83.3)	(13.2–53.5)	(91.4–100)	(1.76–4.90)	(0.03–1.12)

Se: sensitivity; Sp: Specificity; PPV: positive predictive value; NPV: negative predictive value; PLR: positive likelihood ratio; NLR: negative likelihood ratio; CFU: Colony-forming units; ETA: endotracheal aspirate. A likelihood ratio is a probability that a subject with a positive (or negative) test has the disease in question. If the PLR is over 5 and the NLR is under 0.10, it may be concluded that it is essential for a diagnosis and of high validity for use in routine clinical practice. A likelihood ratio of 2.0 corresponds to an approximately +15% increase in probability, which is considered a slight increase. A likelihood ratio of 5.0 corresponds to an approximately +30% increase in probability, which is considered a moderate increase.

## References

[1] Chastre J., Fagon J.Y. (2002). Ventilator-associated pneumonia. Am. J. Respir. Crit. Care Med..

[2] Vincent J.L., Rello J., Marshall J., Silva E., Anzueto A., Martin C.D., Moreno R., Lipman J., Gomersall C., Sakr Y. (2009). International study of the prevalence and outcomes of infection in intensive care units. JAMA.

[3] Reporting on 2011 Surveillance Data and 2012 Epidemic Intelligence Data. Annual Epidemiological Report 2013. Stockholm: European Centre for Disease Prevention and Control (ECDC), 2013. https://ecdc.europa.eu/sites/portal/files/media/en/publications/Publications/annual-epidemiological-report-2013.pdf.

[4] Planquette B., Timsit J.F., Misset B.Y., Schwebel C., Azoulay E., Adrie C., Vesin A., Jamali S., Zahar J.R., Allaouchiche B. (2013). *Pseudomonas aeruginosa* ventilator-associated pneumonia. predictive factors of treatment failure. Am. J. Respir. Crit. Care Med..

[5] Kollef M.H., Hamilton C.W., Ernst F.R. (2012). Economic impact of ventilator-associated pneumonia in a large matched cohort. Infect. Control Hosp. Epidemiol..

[6] Warren D.K., Shukla S.J., Olsen M.A., Kollef M.H., Hollenbeak C.S., Cox M.J., Cohen M.M., Fraser V.J. (2003). Outcome and attributable cost of ventilator-associated pneumonia among intensive care unit patients in a suburban medical center. Crit. Care Med..

[7] Kollef M.H., Burnham C.D. (2017). Ventilator-Associated Pneumonia: The Role of Emerging Diagnostic Technologies. Semin. Respir. Crit. Care Med..

[8] Valencia A.M., Torres M.A., Insausti O.J., Alvarez L.F., Carrasco J.N., Herranz C.M., Tirapu Leon J.P. (2003). Diagnostic value of quantitative cultures of endotracheal aspirate in ventilator-associated pneumonia: A Multicenter Study. Arch. Bronconeumol..

[9] Morris A.C., Kefala K., Simpson A.J., Wilkinson T.S., Everingham K., Kerslake D., Raby S., Laurenson I.F., Swann D.G., Walsh T.S. (2009). Evaluation of the effect of diagnostic methodology on the reported incidence of ventilator-associated pneumonia. Thorax.

[10] Torres A., Fernandez-Barat L. (2014). New developments in the diagnosis of VAP make bronchoalveolar lavage less useful: Some considerations. Intensive Care Med..

[11] Correa R.A., Luna C.M., Anjos J.C., Barbosa E.A., Rezende C.J., Rezende A.P., Pereira F.H., Rocha M.O. (2014). Quantitative culture of endotracheal aspirate and BAL fluid samples in the management of patients with ventilator-associated pneumonia: A randomized clinical trial. J. Bras. Pneumol..

[12] Ruiz M. (2006). A randomized trial of diagnostic techniques for ventilator-associated pneumonia. N. Engl. J. Med..

[13] Ruiz M., Torres A., Ewig S., Marcos M.A., Alcón A., Lledó R., Asenjo M.A., Maldonado A. (2000). Noninvasive versus invasive microbial investigation in ventilator-associated pneumonia: Evaluation of Outcome. Am. J. Respir. Crit. Care Med..

[14] Rea-Neto A., Youssef N.C., Tuche F., Brunkhorst F., Ranieri V.M., Reinhart K., Sakr Y. (2008). Diagnosis of ventilator-associated pneumonia: A Systematic Review of the Literature. Crit. Care.

[15] Landry R.M., An D., Hupp J.T., Singh P.K., Parsek M.R. (2006). Mucin-*Pseudomonas aeruginosa* interactions promote biofilm formation and antibiotic resistance. Mol. Microbiol..

[16] Worlitzsch D., Tarran R., Ulrich M., Schwab U., Cekici A., Meyer K.C., Birrer P., Bellon G., Berger J., Weiss T. (2002). Effects of reduced mucus oxygen concentration in airway *Pseudomonas* infections of cystic fibrosis patients. J. Clin. Investig..

[17] Gries D.M., Pultz N.J., Donskey C.J. (2005). Growth in cecal mucus facilitates colonization of the mouse intestinal tract by methicillin-resistant *Staphylococcus aureus*. J. Infect. Dis..

[18] Hall-Stoodley L., Stoodley P. (2009). Evolving concepts in biofilm infections. Cell Microbiol..

[19] Costerton J.W., Stewart P.S., Greenberg E.P. (1999). Bacterial biofilms: A Common Cause of Persistent Infections. Science.

[20] Trampuz A., Piper K.E., Jacobson M.J., Hanssen A.D., Unni K.K., Osmon D.R., Mandrekar J.N., Cockerill F.R., Steckelberg J.M., Greenleaf J.F. (2007). Sonication of removed hip and knee prostheses for diagnosis of infection. N. Engl. J. Med..

[21] Hoiby N., Bjarnsholt T., Moser C., Bassi G.L., Coenye T., Donelli G., Hall-Stoodley L., Hola V., Imbert C., Kirketerp-Moller K. (2015). ESCMID guideline for the diagnosis and treatment of biofilm infections. Clin. Microbiol. Infect..

[22] Marsh R.L., Thornton R.B., Smith-Vaughan H.C., Richmond P., Pizzutto S.J., Chang A.B. (2015). Detection of biofilm in bronchoalveolar lavage from children with non-cystic fibrosis bronchiectasis. Pediatr. Pulmonol..

[23] Fernandez-Barat L., Li B.G., Ferrer M., Bosch A., Calvo M., Vila J., Gabarrus A., Martinez-Olondris P., Rigol M., Esperatti M. (2012). Direct analysis of bacterial viability in endotracheal tube biofilm from a pig model of methicillin-resistant *Staphylococcus aureus* pneumonia following antimicrobial therapy. FEMS Immunol. Med. Microbiol..

[24] Li B.G., Fernandez-Barat L., Saucedo L., Giunta V., Marti J.D., Tavares R.O., Aguilera X.E., Rigol M., Roca I., Munoz L. (2015). Endotracheal tube biofilm translocation in the lateral Trendelenburg position. Crit. Care.

[25] Martinez-Olondris P., Sibila O., Agusti C., Rigol M., Soy D., Esquinas C., Piner R., Luque N., Guerrero L., Quera M.A. (2010). An experimental model of pneumonia induced by methicillin-resistent *Staphylococcus aureus* in ventilated piglets. Eur. Respir. J..

[26] Li B.G., Rigol M., Marti J.D., Saucedo L., Ranzani O.T., Roca I., Cabanas M., Munoz L., Giunta V., Luque N. (2014). A novel porcine model of ventilator-associated pneumonia caused by oropharyngeal challenge with *Pseudomonas aeruginosa*. Anesthesiology.

[27] Sibila O., Agusti C., Torres A., Baquero S., Gando S., Patron J.R., Morato J.G., Goffredo D.H., Bassi N., Luna C.M. (2007). Experimental *Pseudomonas aeruginosa* pneumonia: Evaluation of the associated inflammatory response. Eur. Respir. J..

[28] American Association for Respiratory Care (2010). Endotracheal suctioning of mechanically ventilated patients with artificial airways. Respir. Care.

[29] Fernandez-Barat L., Ciofu O., Kragh K.N., Pressler T., Johansen U., Motos A., Torres A., Hoiby N. (2017). Phenotypic shift in *Pseudomonas aeruginosa* populations from cystic fibrosis lungs after 2-week antipseudomonal treatment. J. Cyst. Fibros..

[30] Ciofu O., Fussing V., Bagge N., Koch C., Hoiby N. (2001). Characterization of paired mucoid/non-mucoid *Pseudomonas aeruginosa* isolates from Danish cystic fibrosis patients: Antibiotic resistance, beta-lactamase activity and RiboPrinting. J. Antimicrob. Chemother..

[31] Hola V., Ruzicka F., Horka M. (2010). Microbial diversity in biofilm infections of the urinary tract with the use of sonication techniques. FEMS Immunol. Med. Microbiol..

[32] Stickler D.J. (2008). Bacterial biofilms in patients with indwelling urinary catheters. Nat. Clin. Pract. Urol..

[33] Fernandez-Barat L., Ferrer M., Sierra J.M., Soy D., Guerrero L., Vila J., Li B.G., Cortadellas N., Martinez-Olondris P., Rigol M. (2012). Linezolid limits burden of methicillin-resistant *Staphylococcus aureus* in biofilm of tracheal tubes. Crit. Care Med..

[34] Marquette C.H., Copin M.C., Wallet F., Neviere R., Saulnier F., Mathieu D., Duroche A., Ramon P., Tonnel A.B. (1995). Diagnostic tests for pneumonia in ventilated patients: Prospective evaluation of diagnostic accuracy using histology as a diagnostic gold standard. Am. J. Respir. Crit. Care Med..

[35] Jourdain B., Joly-Guillou M.L., Dombret M.C., Calvat S., Trouillet J.L., Gibert C., Chastre J. (1997). Usefulness of quantitative cultures of BAL fluid for diagnosing nosocomial pneumonia in ventilated patients. Chest.

[36] Burillo A., Marin M., Cercenado E., Ruiz-Carrascoso G., Perez-Granda M.J., Oteo J., Bouza E. (2016). Evaluation of the Xpert Carba-R (Cepheid) Assay Using Contrived Bronchial Specimens from Patients with Suspicion of Ventilator-Associated Pneumonia for the Detection of Prevalent Carbapenemases. PLoS ONE.

[37] Souza L.C.D., Mota V.B.R.D., Carvalho A.V.D.S., Correa R.D.G.C., Liberio S.A., Lopes F.F. (2017). Association between pathogens from tracheal aspirate and oral biofilm of patients on mechanical ventilation. Braz. Oral Res..

[38] Garcia-Nunez M., Marti S., Puig C., Perez-Brocal V., Millares L., Santos S., Ardanuy C., Moya A., Linares J., Monso E. (2017). Bronchial microbiome, PA biofilm-forming capacity and exacerbation in severe COPD patients colonized by *P. aeruginosa*. Future Microbiol..

[39] Kalil A.C., Metersky M.L., Klompas M., Muscedere J., Sweeney D.A., Palmer L.B., Napolitano L.M., O’Grady N.P., Bartlett J.G., Carratala J. (2016). Management of Adults With Hospital-Acquired and Ventilator-Associated Pneumonia: Clinical Practice Guidelines by the Infectious Diseases Society of America and the American Thoracic Society. Clin. Infect. Dis..

